# Eugenol: A Potential Modulator of Human Platelet Activation and Mouse Mesenteric Vascular Thrombosis via an Innovative cPLA2-NF-κB Signaling Axis

**DOI:** 10.3390/biomedicines12081689

**Published:** 2024-07-29

**Authors:** Yi Chang, Chih-Wei Hsia, Kuan-Rau Chiou, Ting-Lin Yen, Thanasekaran Jayakumar, Joen-Rong Sheu, Wei-Chieh Huang

**Affiliations:** 1 Department of Anesthesiology, Shin Kong Wu Ho-Su Memorial Hospital, Taipei 111, Taiwan; 2 Graduate Institute of Medical Sciences, College of Medicine, Taipei Medical University, Taipei 110, Taiwan; 3 School of Medicine, College of Medicine, Fu Jen Catholic University, New Taipei City 242, Taiwan; 4 Department of Medical Research, Taipei Medical University Hospital, Taipei 110, Taiwan; 5 Division of Cardiology, Department of Internal Medicine, Shuang Ho Hospital, Taipei Medical University, New Taipei City 235, Taiwan; 6 Department of Medical Research, Cathay General Hospital, Taipei 106, Taiwan; 7 Department of Ecology and Environmental Sciences, Pondicherry University, Puducherry 605014, India; 8 Department of Pharmacology, School of Medicine, College of Medicine, Taipei Medical University, Taipei 110, Taiwan

**Keywords:** eugenol, cPLA2, NF-κB, [Ca^2+^]i, human platelets, platelet plug formation

## Abstract

Background: Platelets, a type of anucleated cell, play a crucial role in cardiovascular diseases (CVDs). Therefore, targeting platelet activation is essential for mitigating CVDs. Endogenous agonists, such as collagen, activate platelets by initiating signal transduction through specific platelet receptors, leading to platelet aggregation. Eugenol, primarily sourced from clove oil, is known for its antibacterial, anticancer, and anti-inflammatory properties, making it a valuable medicinal agent. In our previous study, eugenol was shown to inhibit platelet aggregation induced by collagen and arachidonic acid. We concluded that eugenol exerts a potent inhibitory effect on platelet activation by targeting the PLCγ2–PKC and cPLA2-TxA2 pathways, thereby suppressing platelet aggregation. In our current study, we found that eugenol significantly inhibits NF-κB activation. This led us to investigate the relationship between the NF-κB and cPLA2 pathways to elucidate how eugenol suppresses platelet activation. Methods: In this study, we prepared platelet suspensions from the blood of healthy human donors to evaluate the inhibitory mechanisms of eugenol on platelet activation. We utilized immunoblotting and confocal microscopy to analyze these mechanisms in detail. Additionally, we assessed the anti-thrombotic effect of eugenol by observing fluorescein-induced platelet plug formation in the mesenteric microvessels of mice. Results: For immunoblotting and confocal microscopy studies, eugenol significantly inhibited NF-κB-mediated signaling events stimulated by collagen in human platelets. Specifically, it reduced the phosphorylation of IKK and p65 and prevented the degradation of IκBα. Additionally, CAY10502, a cPLA2 inhibitor, significantly reduced NF-κB-mediated signaling events. In contrast, BAY11-7082, an IKK inhibitor, did not affect collagen-stimulated cPLA2 phosphorylation. These findings suggest that cPLA2 acts as an upstream regulator of NF-κB activation during platelet activation. Furthermore, both BAY11-7082 and CAY10502 significantly reduced the collagen-induced rise in intracellular calcium levels. In the animal study, eugenol demonstrated potential as an anti-thrombotic agent by significantly reducing platelet plug formation in fluorescein-irradiated mouse mesenteric microvessels. Conclusion: Our study uncovered a novel pathway in platelet activation involving the cPLA2-NF-κB axis, which plays a key role in the antiplatelet effects of eugenol. These findings suggest that eugenol could serve as a valuable and potent prophylactic or therapeutic option for arterial thrombosis.

## 1. Introduction

Natural medicinal products have substantially contributed to the advancement of pharmaceuticals, addressing a diverse range of conditions such as cardiovascular diseases (CVDs), cancer, infectious disorders, and more. Eugenol, initially discovered as an aromatic constituent isolated from *Eugenia caryophyllus*, commonly known as clove oil, constitutes a phenolic aromatic compound [1]. Its versatile applications include serving as a topical analgesic and local anesthetic. Eugenol also demonstrates markedly cardiovascular properties, in addition to its analgesic and localized anesthetic effects. Its multifaceted therapeutic potential positions eugenol as a promising candidate for the development of novel pharmaceuticals targeting various cardiovascular disease risk factors through distinct pathways. For instance, studies have demonstrated eugenol’s ability to induce hypotensive and vasorelaxant effects via diverse mechanisms, including the inhibition of the angiotensin-converting enzyme, the activation of endothelial nitric oxide synthase, the inhibition of calcium influx, and the activation of potassium channels [2]. These effects hold therapeutic significance for the treatment of CVDs.

CVDs, such as coronary artery disease, stroke, and hypertension, rank as the foremost cause of death globally. Platelets play a critical role in CVDs by forming blood clots, a normal response to injury. However, in conditions like atherosclerosis, where arterial plaques accumulate, platelets can become activated and contribute to clot formation within these vessels. This platelet aggregation often leads to arterial thrombosis, a common complication of CVDs that can result in serious events such as heart attacks or strokes [3]. Consequently, antiplatelet medications like aspirin and clopidogrel are frequently prescribed to inhibit platelet aggregation, reducing the risk of thrombosis and preventing cardiovascular events in high-risk individuals [4,5,6]. However, the effectiveness of these medications is frequently impeded by adverse effects. Aspirin can cause complications like gastric ulcers and bleeding, while clopidogrel is linked to conditions such as aplastic anemia and thrombocytopenic purpura [7,8]. Therefore, there is a critical need for new therapeutic agents that provide better safety and efficacy in managing and preventing CVDs, ideally with minimal or no adverse drug effects.

Platelets, anucleated cells originating from megakaryocytes [9], typically remain inactive in the bloodstream until they are triggered by intraluminal thrombosis. When an injury occurs, platelets adhere to the damaged vascular lining, releasing bioactive compounds and clustering together. Collagen is crucial in this process, as it interacts with collagen receptors, facilitating platelet adhesion and activation. Eugenol has demonstrated a wide range of notable pharmacological activities; however, there is limited research addressing its specific impact on platelet activation, particularly in human subjects. In our recent study, we observed significant efficacy in the inhibition of human platelet aggregation induced by collagen and arachidonic acid (AA) with eugenol at concentrations ranging from 1 to 4 μM. However, even at concentrations up to 100 µM, eugenol did not inhibit aggregation induced by thrombin or U46619. Based on these findings, we concluded that eugenol exerts potent inhibitory effects by targeting the phospholipase Cγ2 (PLCγ2)–protein kinase C (PKC) and cytosolic phospholipase A2 (cPLA2)–thromboxane A2 (TxA2) cascade, ultimately suppressing platelet aggregation [10]. In our current study, we observed that eugenol significantly inhibits nuclear factor-κB (NF-κB) activation. This discovery prompted us to further explore the relationship between NF-κB and other signaling pathways, aiming to elucidate the detailed mechanism by which eugenol suppresses platelet activation. As previously reported [10], eugenol significantly decreased mortality in mice with ADP-induced acute pulmonary thromboembolism. This study further investigates eugenol’s anti-thrombotic potential using a different animal model, focusing on sodium fluorescein-irradiated platelet plug formation in mouse mesenteric microvessels.

## 2. Materials and Methods

### 2.1. Reagents and Materials

Eugenol (≥98.5%) was purchased from MedChem Express (Monmouth Junction, NJ, USA). Collagen (type I), sodium pyrophosphate, sodium orthovanadate, leupeptin, sodium fluoride (NaF), aprotinin, phenylmethylsulfonyl fluoride (PMSF), bovine serum albumin (BSA), and ethylenediaminetetraacetic acid (EDTA) were purchased from Sigma (St. Louis, MO, USA). Anti-IκBα (44D4) monoclonal antibody (mAb) was purchased from Cell Signaling (Beverly, MA, USA). Phospho-c-PLA2 (Ser^505^), phospho-NFκB p65 (Ser^536^), and phospho-IKK alpha/beta (Ser^180^/Ser^181^) polyclonal antibodies (pAbs) were purchased from Affinity (Cincinnati, OH, USA). Protein assay dye reagent concentrate was purchased from Bio-Rad Laboratories Inc. (Hercules, CA, USA). Fura-2-acetoxymethyl ester (Fura 2-AM) was purchased from Molecular Probes (Eugene, OR, USA). CF^TM^488A Dye, CF^TM^647M, and isotype control IgG Dye were obtained from Biotium (Hayward, CA, USA). Anti-α-tubulin mAb was purchased from NeoMarkers (Fremont, CA, USA). Amersham (Buckinghamshire, UK) supplied Hybond-P polyvinylidene difluoride membranes, enhanced chemiluminescence Western blotting detection reagent, horseradish peroxidase-conjugated donkey anti-rabbit immunoglobulin G (IgG), and sheep anti-mouse IgG. A 0.1% dimethyl sulfoxide (DMSO) was used to dissolve eugenol, and the stock solution was stored at 4 °C.

### 2.2. Preparation of Human Washed Platelets and Analysis of the Changes of [Ca^2+^]i Level

Approval for this study was obtained from the Institutional Review Board of Taipei Medical University (TMU-JIRB-N202212035), in compliance with the ethical principles outlined in the Helsinki Declaration. This approval ensured that all aspects of the study adhered to internationally recognized ethical standards for conducting research involving human participants. Informed consent was acquired from all human blood donors who participated in the study by completing a consent form before enrollment. This process involved providing the donors with detailed information about the study’s purpose, procedures, potential risks, and benefits, ensuring that they fully understood and voluntarily agreed to participate. The preparation of platelet suspensions from healthy human donor blood meticulously followed a previously described method [10]. This method involved combining whole blood with an acid-citrate–dextrose solution (in a ratio of 9:1, *v*/*v*) and subjecting it to subsequent centrifugation steps to isolate platelet-rich plasma (PRP). The PRP was then supplemented with EDTA (2 mM) and heparin (6.4 U/mL). After a brief incubation period, another round of centrifugation was conducted, followed by the resuspension and additional centrifugation of the resulting platelet pellets. These pellets were then suspended in Tyrode’s solution enriched with BSA at a concentration of 3.5 mg/mL and Ca^2+^ at 1 mM. Platelet counts were determined using a Coulter counter (Beckman Coulter, Miami, FL, United States). To evaluate intracellular calcium mobilization ([Ca^2+^]i), platelet suspensions (3.6 × 10^8^ cells/mL) were incubated with 0.1% DMSO, eugenol, or test compounds in the presence of Fura 2-AM (5 μM). The levels of Fura 2-AM were quantified using a Hitachi Spectrometer F-7000 (Tokyo, Japan) with excitation wavelengths of 340 nm and 380 nm, and an emission wavelength of 500 nm.

### 2.3. Immunoblotting

The washed platelets (1.2 × 10^9^ cells/mL) were incubated with either 0.1% DMSO, eugenol, or a selection of test compounds to assess their effects. After this initial incubation, the platelets were subjected to stimulation with or without collagen for a duration of 5 min to simulate physiological activation. For the purpose of subsequent analysis, a specialized 200 μL lysis buffer was prepared, containing a mixture of protease inhibitors and other reagents, as follows: aprotinin (10 μg/mL), sodium orthovanadate (1 mM), PMSF (1 mM), NaF (10 mM), leupeptin (2 μg/mL), and sodium pyrophosphate (5 mM). This lysis buffer was then added to the platelets, which were resuspended thoroughly in the buffer and allowed to incubate for 1 h to ensure complete cell lysis. Following the lysis incubation, the samples were centrifuged at 5000× *g* for 5 min to separate the cellular debris from the supernatant. The supernatant, now containing the protein lysates, was carefully collected without disturbing the pellet. The protein concentrations were quantified using the Bradford protein assay (Bio-Rad, Hercules, CA, USA). An aliquot containing 80 μg of protein was taken for further processing. The protein samples were subjected to separation using 8% SDS-PAGE. To identify and analyze specific target proteins within the lysates, the corresponding primary antibodies were employed. These antibodies bind to their target proteins with high specificity, allowing for precise detection. The optical density of the resulting protein bands was then quantified using a video densitometer, which measures the intensity of the bands. This quantification process was facilitated by the use of Bio-profil Biolight software, Version V2000.01 (Vilber Lourmat, Marne-la-Vallée, France). Relative protein expression was determined by normalizing the expression levels to the total protein content of interest.

### 2.4. Exploring Confocal Laser Fluorescence Microscopy: Unveiling Cellular Insights

Resting or collagen-activated platelets were carefully immobilized on poly-L-lysine-coated coverslips, followed by fixation in a solution containing 4% (*v*/*v*) paraformaldehyde for 1 h. After fixation, platelets underwent permeabilization using 0.1% Triton X-100 and were subsequently incubated in a 5% BSA solution in phosphate-buffered saline (PBS) for 1 h to block nonspecific binding sites effectively. Following this preparation, platelets were subjected to immunostaining with specific primary antibodies targeting the proteins of interest over a 24 h period. After immunostaining, thorough washing with PBS was carried out, and the platelets were then exposed to secondary antibodies (Alexa Fluor^®^ 488-labeled goat anti-rabbit IgG and Alexa Fluor^®^ 647-labeled goat anti-mouse IgG) for an additional hour. Finally, the imaging of the platelets was conducted using a confocal microscope (Leica TCS SP5, Mannheim, Germany) equipped with a 100× oil immersion objective.

### 2.5. Evaluating Sodium Fluorescein-Induced Vascular Thrombosis in Mouse Mesenteric Microvessels

Prior to commencing in vivo experiments, this study received approval from the Institutional Animal Care and Use Committee of Taipei Medical University (Approval ID: LAC-2022-0320). A total of 48 male ICR mice were then divided into four groups, each comprising 12 mice: (1) sham, (2) DMSO-treated (0.1%, i.p.), (3) eugenol-treated (6 mg/kg, i.p.), and (4) eugenol-treated (15 mg/kg, i.p.). Subsequently, sodium fluorescein (15 μg/kg) was administered intravenously, following the protocol outlined in a previous study [11]. Anesthesia was induced in the mice using intraperitoneal sodium pentobarbital (50 mg/kg). Venules (30–40 µm) were then exposed to irradiation at a wavelength of <520 nm to induce microthrombus formation, and the time taken for the thrombus to occlude the microvessel (referred to as “occlusion time”) was recorded.

### 2.6. Statistical Analysis

The data are expressed as mean ± standard error of the mean (SEM). The value of n represents the number of independent experiments conducted using blood samples obtained from distinct donors. To assess significant differences among the experimental groups, we employed one-way analysis of variance (ANOVA) followed by the Student–Newman–Keuls post hoc test to mitigate family wise type I error. A *p*-value of <0.05 was considered statistically significant. All statistical analyses were carried out using SAS software (version 9.2; SAS, Cary, NC, USA).

## 3. Results

### 3.1. Assessing the Effects of Eugenol on Platelet NF-κB Activation Stimulated by Collagen

**Figure 1:**
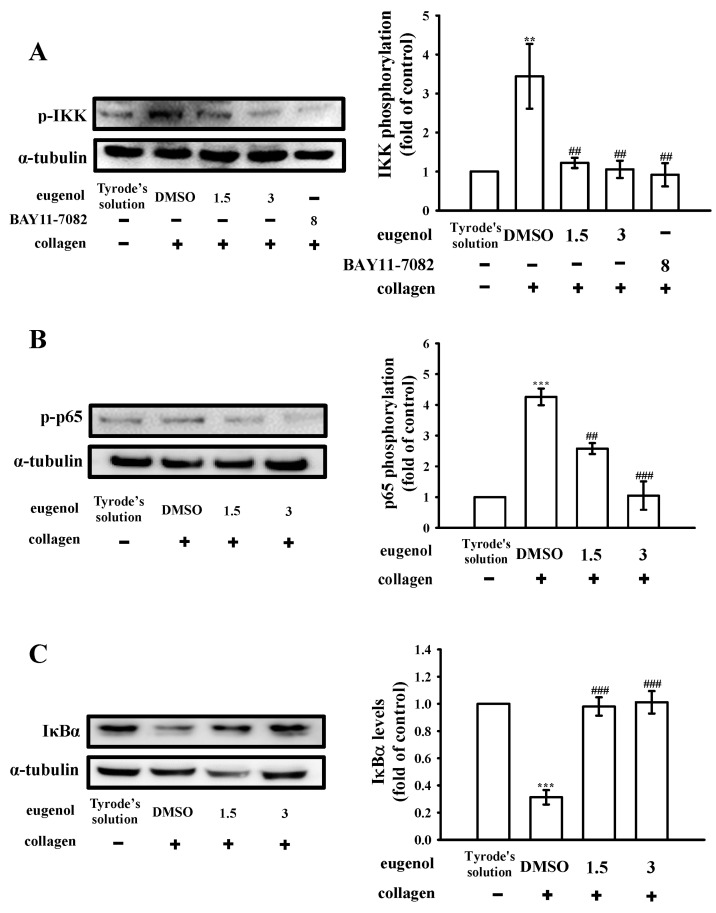
The effect of eugenol on the regulation of NF-κB signaling in platelets. Washed platelets (1.2 × 10^9^ cells/mL) were either incubated with Tyrode’s solution alone or preincubated with 0.1% DMSO, BAY11-7082 (8 µM), or eugenol (1.5 and 3 µM), followed by the addition of collagen (1 μg/mL) to induce (**A**) IKK and (**B**) p65 phosphorylation, as well as (**C**) IκBα degradation for immunoblotting analysis. The relevant statistical data are displayed in the right panel of each figure. Data are expressed as the mean ± standard error of the mean (*n* = 4). ** *p* < 0.01 and *** *p* < 0.001 denote significance compared with the resting (Tyrode’s solution) group; ^##^ *p* < 0.01 and ^###^ *p* < 0.001 indicate significance compared with the 0.1% DMSO + collagen group.

In our previous study [10], we observed that eugenol exerts a potent inhibitory effect on platelet activation by targeting the PLCγ2–PKC and cPLA2–TxA2 cascade. This inhibition subsequently leads to the suppression of the phosphoinositide 3-kinase (PI3K)-Akt and mitogen-activated protein kinase (MAPK) signaling pathways. Building upon these findings, our present study aims to delve deeper into the involvement of the NF-κB signaling pathway in eugenol-mediated antiplatelet activation. NF-κB is a protein complex that holds pivotal significance in the regulation of genes associated with inflammation, immune responses, cell proliferation, survival, and platelet activation [12]. Typically, NF-κB is present in the cytoplasm as an inactive complex consisting of p50 and p65 subunits, which are tightly bound to the inhibitor κB (IκB) [13]. Upon the administration of collagen (1 μg/mL), we observed the enhanced phosphorylation of IκB kinase (IKK) and p65, along with the degradation of IκBα (Figure 1A–C). However, treatment with eugenol at concentrations of 1.5 and 3 μM resulted in a clear reduction in IKK and p65 phosphorylation, and a reversal of IκBα degradation, following collagen stimulation. Moreover, BAY11-7082, an IKK inhibitor belonging to the family of NF-κB inhibitors, has been extensively employed in various applications including anti-cancer, anti-inflammatory, and neuroprotective activities [14]. In our current investigation, it was observed that pre-exposure to BAY11-7082 (8 µM) yielded notable inhibitory effects on IKK phosphorylation induced by collagen stimulation, as depicted in Figure 1A. The statistical analysis of these results is presented in the panels to the right of the corresponding Western blot images, as depicted in Figure 1A–C. These findings strongly suggest that the antiplatelet activity of eugenol is closely associated with the inhibition of NF-κB signaling pathways.

**Figure 2:**
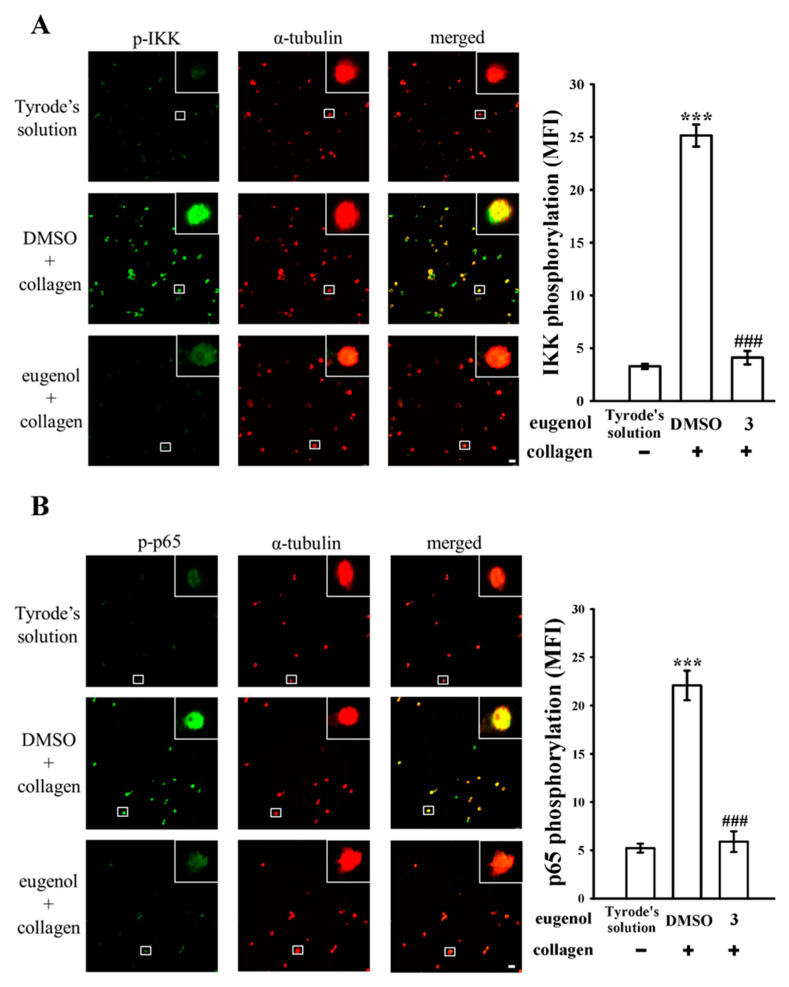
The effect of eugenol on the regulation of NF-κB signaling observed via confocal microscopy. Washed platelets (1.2 × 10^9^ cells/mL) were either incubated with Tyrode’s solution alone or preincubated with 0.1% DMSO or eugenol (3 µM), followed by the addition of collagen (1 μg/mL) for confocal microscopic assessment. This examination aimed to visualize phosphorylated (**A**) IKK and (**B**) p65 through green fluorescence, and α-tubulin through red fluorescence. For this purpose, Alexa Fluor^®^ 488-labeled goat anti-rabbit IgG and Alexa Fluor^®^ 647-labeled goat anti-mouse IgG were utilized. The data include associated statistical analysis (MFI: mean fluorescence intensity). Results are presented as mean ± standard error of the mean (*n* = 4). Significant differences are denoted by *** *p* < 0.001 compared to resting platelets exposed to Tyrode’s solution and ^###^ *p* < 0.001 compared to the 0.1% DMSO + collagen group. Scale bar: 5 μm.

Furthermore, we employed confocal scanning fluorescence microscopy to further investigate the inhibition of NF-κB activation by eugenol. This technique allowed us to visualize the fluorescence signals, with green fluorescence indicating IKK or p65 activation and red fluorescence representing α-tubulin, in both resting and collagen-activated platelets (Figure 2A,B). Upon collagen stimulation, there was a notable increase in the fluorescence intensity of phosphorylated IKK or p65 compared to resting platelets. However, these intensities were markedly reduced in platelets treated with eugenol at a concentration of 3 µM. Notably, no significant difference in α-tubulin intensity was observed among the experimental groups (Figure 2A,B). The statistical analysis of these findings is presented in the right panels of the confocal fluorescence image, as illustrated in Figure 2A,B.

### 3.2. Exploring the Interplay between cPLA2 and NF-κB Signaling in Human Platelets

**Figure 3:**
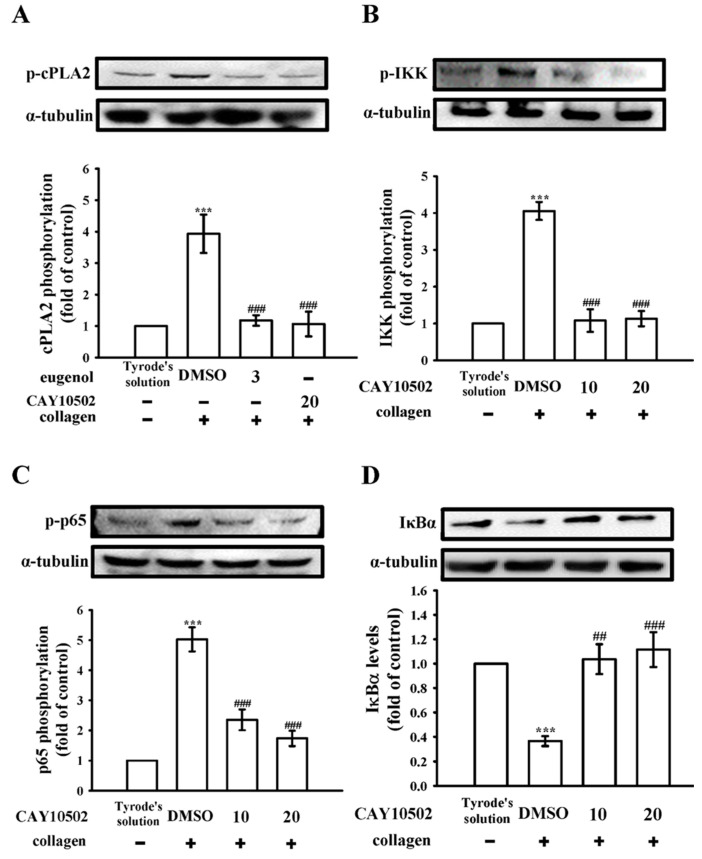
Investigation of CAY10502 on the regulation of cPLA2 and NF-κB signaling in platelets. Washed platelets (1.2 × 10^9^ cells/mL) were either incubated with Tyrode’s solution alone or preincubated with 0.1% DMSO, CAY10502 (20 µM), or eugenol (3 µM), followed by the addition of collagen (1 μg/mL) to induce (**A**) cPLA2, (**B**) IKK, and (**C**) p65 phosphorylation, as well as (**D**) IκBα degradation for immunoblotting analysis. The relevant statistical data are displayed in the right panel of each figure. Data are expressed as the mean ± standard error of the mean (*n* = 4). *** *p* < 0.001 denotes significance compared with the resting (Tyrode’s solution) group; ^##^ *p* < 0.01 and ^###^ *p* < 0.001 indicate significance compared with the 0.1% DMSO + collagen group.

**Figure 4:**
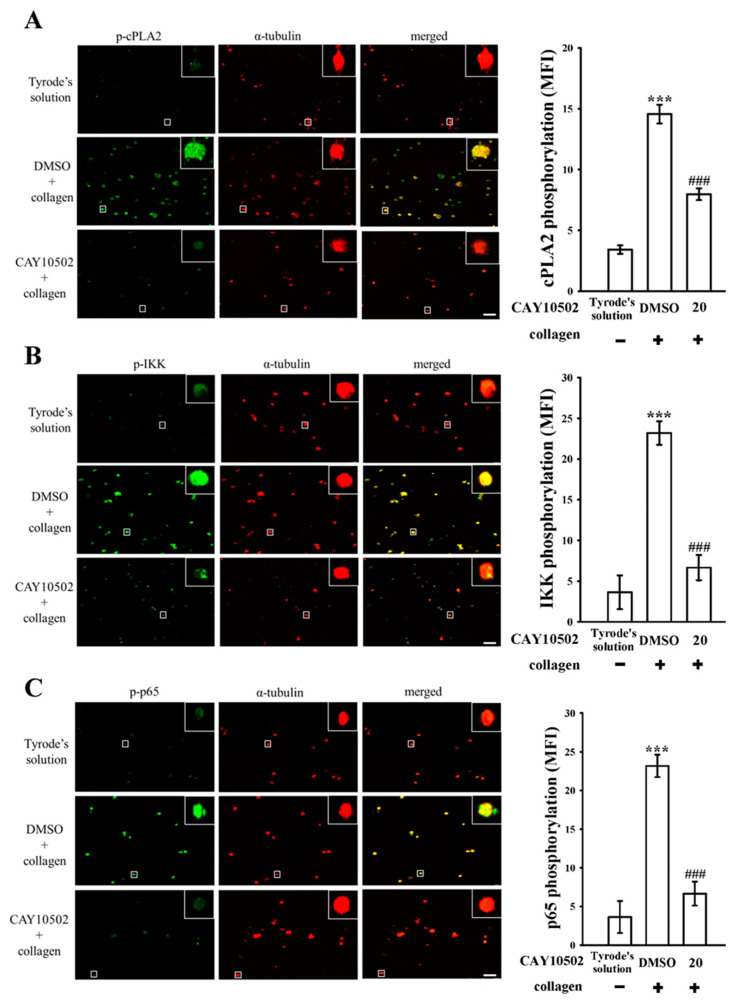
Investigation of CAY10502 on the regulation of cPLA2 and NF-κB signaling observed via confocal microscopy. Washed platelets (1.2 × 10^9^ cells/mL) were either incubated with Tyrode’s solution alone or preincubated with 0.1% DMSO or CAY10502 (20 µM), followed by the addition of collagen (1 μg/mL) for confocal microscopic assessment. This examination aimed to visualize phosphorylated (**A**) cPLA2, (**B**) IKK, and (**C**) p65 through green fluorescence, and α-tubulin through red fluorescence. The data include associated statistical analysis (MFI: mean fluorescence intensity). Results are presented as mean ± standard error of the mean (*n* = 4). Significant differences are denoted by *** *p* < 0.001 compared to resting platelets exposed to Tyrode’s solution and ^###^ *p* < 0.001 compared to the 0.1% DMSO + collagen group. Scale bar: 5 μm.

The enzyme cPLA2 plays a critical role in platelet activation by triggering the release of arachidonic acid (AA), which, in turn, promotes platelet aggregation [15]. Within the scope of our investigation, both eugenol (3 µM) and CAY10502 (20 µM), an inhibitor of cPLA2 [16], demonstrated a noteworthy reduction in cPLA2 phosphorylation in collagen-activated platelets (Figure 3A). In addition, CAY10502 (10 and 20 µM) also exhibited a clear reduction in IKK and p65 phosphorylation, and a reversal of IκBα degradation, following collagen stimulation (Figure 3B–D). To further explore the impact of CAY10502 on NF-κB activation, confocal fluorescence microscopy was utilized. The green fluorescence signals indicate cPLA2, IKK, or p65 activation, while the red fluorescence represents α-tubulin, in both resting and collagen-activated platelets (Figure 4A–C). Upon collagen stimulation, significant increases in fluorescence intensity were observed for phosphorylated cPLA2, IKK, and p65 in comparison to resting platelets (Figure 4A–C). However, treatment with CAY10502 (20 µM) notably reduced these intensities, suggesting a potential regulatory role of cPLA2 in NF-κB activation during platelet activation. There are no significant differences in the fluorescence intensity of cPLA2 between the resting and collagen-activated groups stained with isotype control IgG (please see Figure S1).

### 3.3. Evaluating the Impact of NF-κB on cPLA2 Activation and the Cytosolic Ca^2+^ Mobilization in Platelets

**Figure 5:**
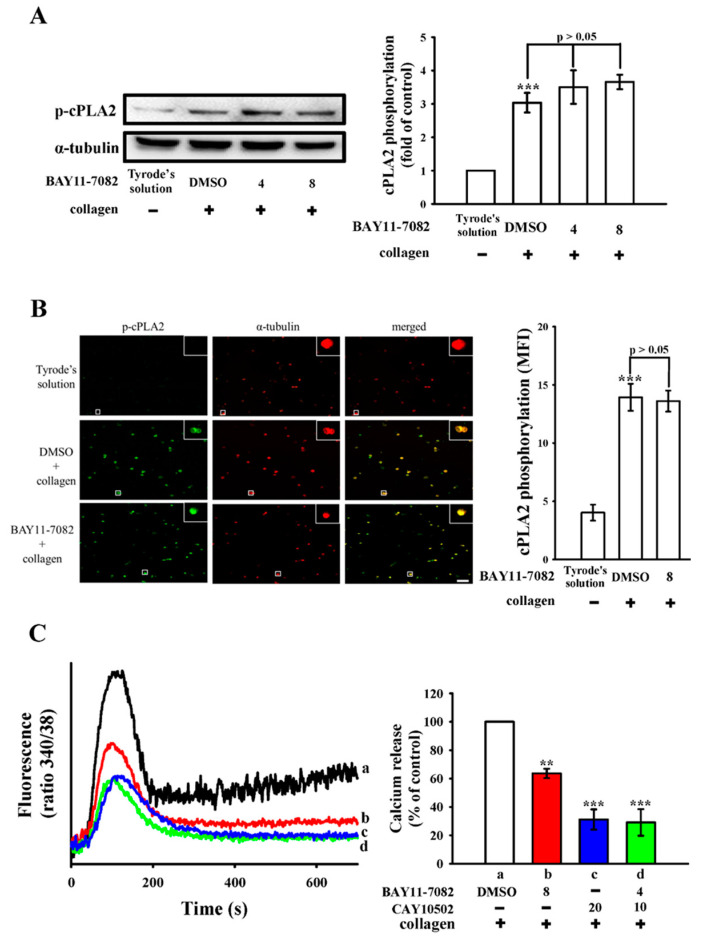
Examination of BAY11-7082 and CAY10502 on cPLA2 phosphorylation and relative [Ca^2+^]i mobilization in human platelets. Washed platelets (1.2 × 10^9^ cells/mL) were either incubated with Tyrode’s solution alone or preincubated with 0.1% DMSO or BAY11-7082 (4 and 8 µM), followed by the addition of collagen (1 μg/mL) to induce cPLA2 phosphorylation for (**A**) immunoblotting analysis and (**B**) confocal microscopic study (green fluorescence for phosphorylated cPLA2 and red fluorescence for α-tubulin). For the study of relative [Ca^2+^]i mobilization, platelet suspensions (3.6 × 10^8^ cells/mL) were incubated with (a) 0.1% DMSO, (b) BAY11-7082 8 µM, (c) CAY10502 20 µM or (d) BAY11-7082 4 µM + CAY10502 10 µM in the presence of Fura 2-AM (5 μM), followed by the addition of collagen (1 μg/mL) to trigger relative [Ca^2+^]i mobilization. Results are presented as mean ± standard error of the mean (*n* = 4). Significant differences are denoted by *** *p* < 0.001 compared to (**A**,**B**) resting platelets exposed to Tyrode’s solution, and (**C**) ** *p* < 0.01 and *** *p* < 0.001 compared to the 0.1% DMSO + collagen group. Scale bar: 5 μm.

As depicted in Figure 5A, pretreatment with BAY11-7082 (8 µM) did not yield notable effects on collagen-induced cPLA2 phosphorylation. Furthermore, consistent outcomes were observed through confocal fluorescence microscopy analysis, as shown in Figure 5B. These findings unequivocally suggest that NF-κB does not serve as an upstream modulator of cPLA2 phosphorylation in platelet activation. Elevated intracellular calcium concentration ([Ca^2+^]i) is known to promote platelet aggregation, a critical step in platelet activation. Fluorescence-based assays were employed to quantify [Ca^2+^]i levels. As demonstrated in Figure 5C, both BAY11-7082 (8 µM) and CAY10502 (20 µM) substantially reduced the collagen-induced increase in [Ca^2+^]i by approximately 35% and 70%, respectively. Remarkably, halving the concentrations of these inhibitors maintained a level of inhibition comparable to that observed with the administration of each inhibitor individually. The statistical evaluation of these observations is delineated in the corresponding panels of Figure 5C.

### 3.4. Evaluating the Anti-Thrombotic Potential of Eugenol in Sodium Fluorescein-Irradiated Mouse Mesenteric Microvessels

**Figure 6:**
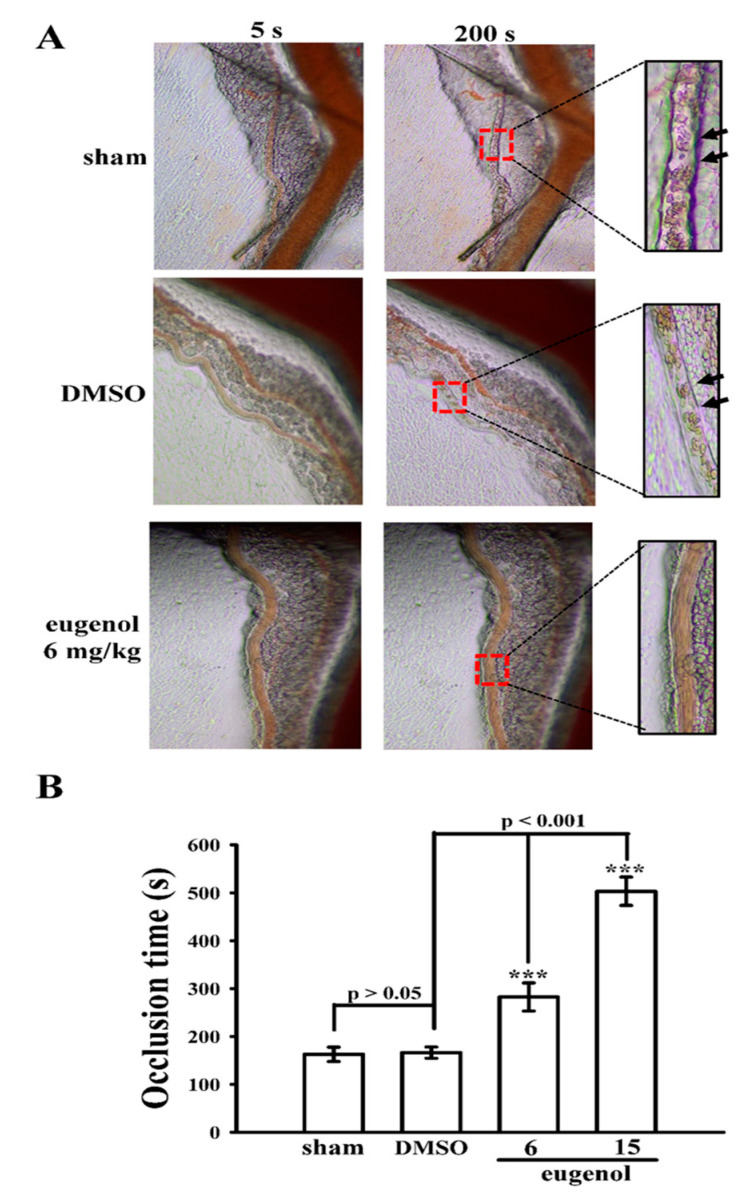
Effect of eugenol on thrombotic platelet plug formation in mesenteric venules in mice. Mice were administered an intraperitoneal injection of normal saline (sham group), solvent control (0.1% DMSO), or eugenol (6 and 15 mg/kg). Mesenteric venules were irradiated with fluorescein to induce microthrombus formation (occlusion time). (**A**) Microscopic images of the sham group, 0.1%, DMSO-treated control, and 6 and 15 mg/kg eugenol-treated groups were recorded at 5 s and 200 s after irradiation. Black arrows indicate platelet plug formation (400× magnification). (**B**) The relevant statistical data are displayed in the bottom panel of the figure. Data are presented as the mean ± standard error of the mean (*n* = 12). *** *p* < 0.001 compared with the 0.1% DMSO-treated group.

As previously reported [10], administering 15 mg/kg of eugenol significantly decreased mortality rates in mice experiencing ADP-induced acute pulmonary thromboembolism. Building upon this prior research, the current study extends the investigation into the anti-thrombotic potential of eugenol using a distinct animal model, focusing on sodium fluorescein-irradiated platelet plug formation within mouse mesenteric microvessels. In mice pretreated with fluorescein sodium (15 µg/kg), the occlusion time in mesenteric microvessels was 163 ± 15 s (*n* = 12) following treatment with normal saline (control sham group) (Figure 6A,B). Treatment with 0.1% DMSO did not significantly extend this occlusion time (166 ± 12 s; *n* = 12), whereas administration of eugenol at doses of 6 and 15 mg/kg notably prolonged occlusion times (6 mg/kg eugenol: 282 ± 29 s; 15 mg/kg eugenol: 503 ± 29 s, *n* = 12; Figure 6B). Following irradiation, thrombotic platelet plug formation was observed in mesenteric microvessels at 200 s but not at 5 s in the 0.1% DMSO-treated group (Figure 6A; indicated by black arrow). However, administration of either 6 or 15 mg/kg eugenol prevented platelet plug formation at both 5 and 200 s post-irradiation (Figure 6A).

## 4. Discussion

Eugenol is a natural and valuable compound with potential applications in various fields, including medicine, dentistry, and food preservation. In our earlier investigation [10], it was observed that eugenol strongly inhibits human platelet activation induced by collagen and AA. This activity is initially mediated through its inhibition of the PLCγ2–PKC and cPLA2–TxA2 cascade. Consequently, downstream signaling pathways including PI3K-Akt and MAPK are suppressed. This multifaceted mechanism ultimately results in the reduction in intracellular calcium concentration ([Ca^2+^]i) and the subsequent inhibition of platelet aggregation. Eugenol exerts a crucial influence among these mechanisms by inhibiting the activation of cPLA2.

Extensive research has delved into the pivotal role of NF-κB within nucleated cells. The activation of NF-κB can be triggered by various stimuli such as free radicals, viral/bacterial infections, or cytokines, resulting in the inflammation and disruption of normal cellular functions [17]. Consequently, NF-κB has emerged as a promising therapeutic target for inflammation-related diseases [18]. Typically residing in the cytoplasm in an inactive state, NF-κB predominantly exists as a heterodimer comprising p50 and p65 subunits, tightly associated with IκBα, the more prevalent of the two subunits (IκBα and IκBβ). Upon activation, NF-κB undergoes a series of events initiated by the phosphorylation of IκBα by the IKK complex. The subsequent dissociation of IκBα from NF-κB subunits ensues, followed by the ubiquitination and rapid degradation of IκBα by proteasomes, facilitating NF-κB nuclear translocation [12]. Notably, research has revealed that although platelets are without a nucleus, they express several transcription factors that have non-genomic functions [19,20]. Moreover, the activation of NF-κB in human atherosclerotic plaques has been linked to the progression of unstable coronary plaques [21]. In the context of platelet biology, NF-κB plays a role in platelet activation, and inhibitors of NF-κB have demonstrated the ability to suppress platelet activation [22].

**Figure 7:**
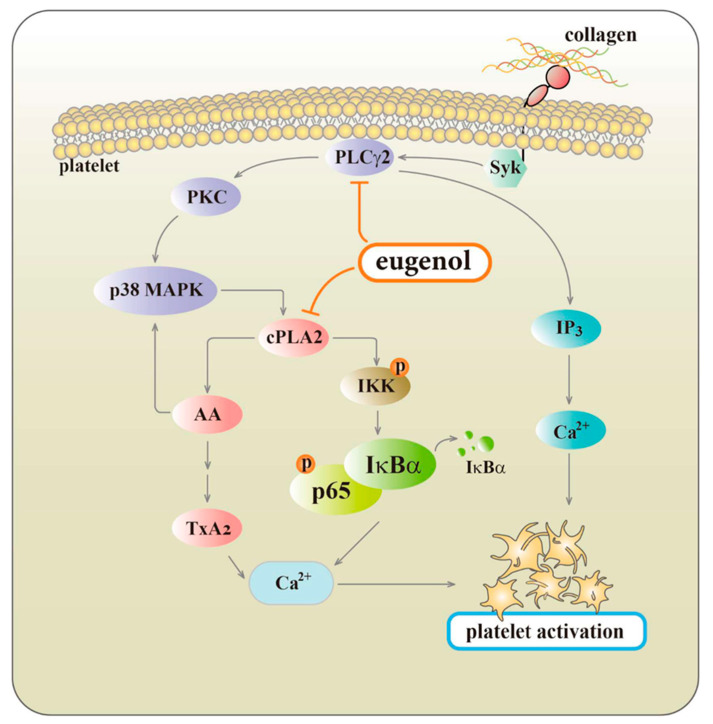
Proposed mechanisms of eugenol’s inhibitory effects on platelet activation. Eugenol impacts key signaling pathways, particularly PLCγ2-PKC and cPLA2-TxA2. Additionally, we identified a novel pathway involving the cPLA2-NF-κB-[Ca^2+^]i axis, which leads to the suppression of platelet aggregation.

Upon the activation of platelets by different stimuli, such as collagen, certain phospholipase enzymes like cPLA2 become activated [23]. These enzymes facilitate the cleavage of AA from phospholipids within the cell membrane. Following this, AA can undergo metabolism by cyclooxygenase to produce TxA2, a crucial mediator that is propelled by the activation of p38 MAPK in reaction to platelet agonists (Figure 7) [10]. This process serves to amplify platelet activation. The interconnected roles of the AA-TxA2 and PLCγ2-PKC pathways in platelet signaling pathways are apparent, with PLCγ2 initiating signaling events and generating second messengers, while AA-TxA2 plays a role in subsequent processes that further enhance platelet activation (Figure 7) [10].

Platelets store various molecules in granules, and [Ca^2+^]i plays a vital role in releasing these contents upon platelet activation. Inhibiting [Ca^2+^]i mobilization disrupts the release of molecules like ADP and serotonin from dense granules, reducing the positive feedback loop that enhances platelet activation and aggregation **[24]**. Additionally, decreased [Ca^2+^]i affects the platelet’s ability to bind fibrinogen, a critical step in aggregation, as Ca^2+^ is essential for activating integrin receptors for tight fibrinogen binding [25]. This impairment leads to decreased platelet aggregation and clot formation. Furthermore, [Ca^2+^]i acts as a secondary messenger in multiple signaling pathways involved in platelet activation. For instance, Ca^2+^ participates in activating PLC, which generates inositol trisphosphate (IP3) and diacylglycerol (DAG). IP3 prompts the release of Ca^2+^ from intracellular stores, amplifying platelet activation (Figure 7). Blocking this process hampers platelet activation [25]. In our study, we observed that cPLA2 regulates NF-κB activation, and both cPLA2 and NF-κB seem to have an additive effect in inhibiting [Ca^2+^]i mobilization during platelet activation (see Figure 5C).

To gauge the effectiveness of therapeutic compounds in treating arterial vascular thrombosis, animal models are indispensable. A mouse model, in particular, should ideally be straightforward and replicable. Throughout the thrombosis study [26], endothelial cell injury was induced in mesenteric venules by continuous irradiation with fluorescein over the experimental duration. Administering eugenol at doses of 6 and 15 mg/kg resulted in a dose-dependent extension of occlusion time. This finding aligns with the well-established understanding that platelet aggregation significantly contributes to the risk of vascular thrombosis. Consequently, this nuanced discovery underscores the potential of eugenol as an effective natural agent for addressing thromboembolic disorders, offering a potentially beneficial substitute for conventional antiplatelet therapies.

## 5. Conclusions

Eugenol, a potent new antithrombotic compound, serves as a natural inhibitor of platelet activation in humans. In our research, we have uncovered a novel signaling pathway involving cPLA2-NF-κB-[Ca^2+^]i axis inhibition by eugenol when stimulated by collagen (Figure 7). However, we acknowledge the possibility of other as-yet-unidentified mechanisms contributing to the eugenol-mediated suppression of platelet activation. The findings from this study highlight the potential therapeutic and preventive uses of eugenol in arterial thrombosis.

## Data Availability

The datasets used and analyzed during the current study are available from the corresponding author on reasonable request due to ethical considerations and the presence of unpublished data.

## Supplementary Materials

The following supporting information can be downloaded at: https://www.mdpi.com/article/10.3390/biomedicines12081689/s1, Figure S1: Confocal image of control IgG and p-cPLA2 in human platelets. The confocal image (10 × 100 magnification) of control IgG or p-cPLA2 in Tyrode’s solution or collagen-activated platelets. Control IgG or p-cPLA2 was labeled with goat anti-rabbit IgG-conjugated FITC (shown in green color) as described in Materials and Methods.

## Abbreviations

AA	arachidonic acid
BSA	bovine serum albumin
cPLA2	cytosolic phospholipase A2
CVDs	cardiovascular diseases
DAG	diacylglycerol
DMSO	dimethyl sulfoxide
EDTA	ethylenediaminetetraacetic acid
IKK	inhibitor of κB kinase
IP3	inositol trisphosphate
IκB	inhibitor of κB
MAPK	mitogen-activated protein kinase
NaF	sodium fluoride
NF-κB	nuclear factor-κB
PI3K	phosphoinositide 3-kinase
PKC	protein kinase C
PLCγ2	phospholipase Cγ2
PMSF	phenylmethylsulfonyl fluoride
PRP	platelet-rich plasma
TxA2	thromboxane A2
U46619	(5Z)-7-[(1R,4S,5S,6R)-6-[(1E,3S)-3-hydroxy-1-octen-1-yl]-2-oxabicyclo [2.2.1]hept-5-yl]-5-heptenoic acid
