# Supplementary material for: Eugenol: A Potential Modulator of Human Platelet Activation and Mouse Mesenteric Vascular Thrombosis via an Innovative cPLA2-NF-κB Signaling Axis
Source: Biomedicines. 2024 Jul 29;12(8):1689. doi: 10.3390/biomedicines12081689 (PMC11351298; doi:10.3390/biomedicines12081689)
Supplement: Supplementary file 1 [file biomedicines-12-01689-s001.zip › biomedicines-3097053-supplementary.pdf]

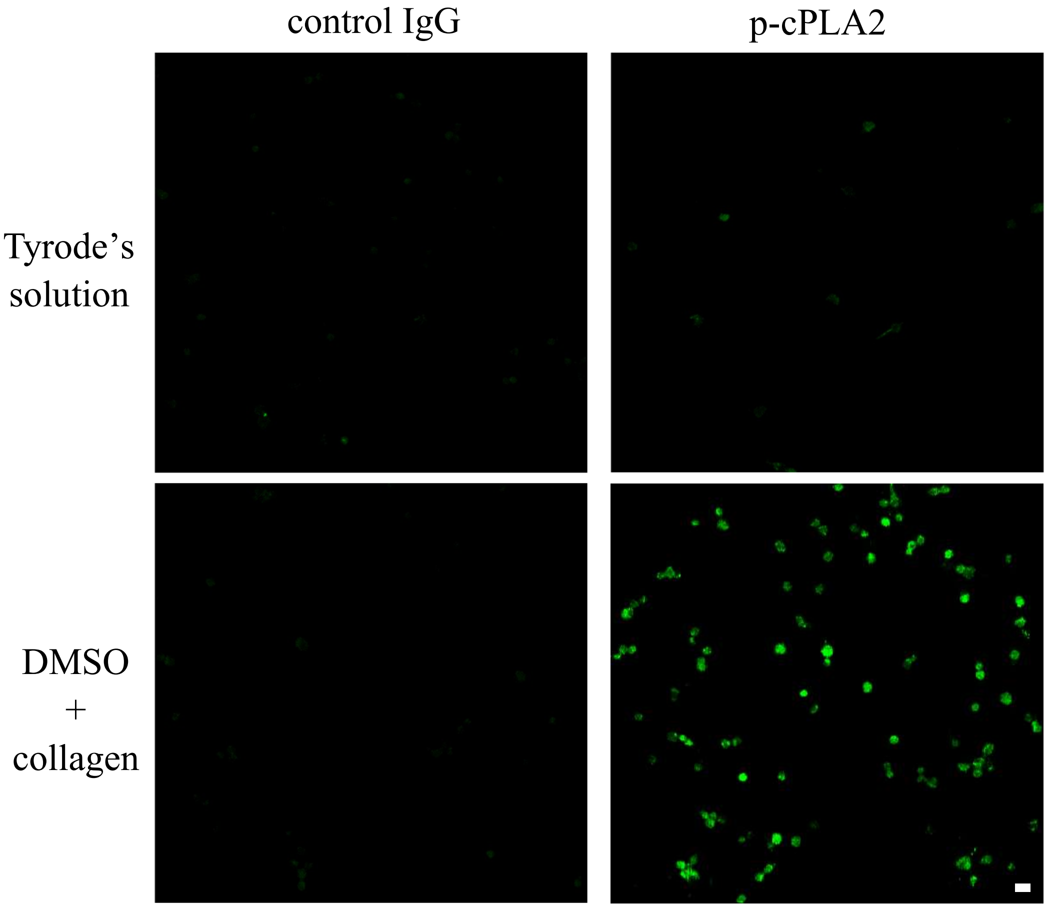

Figure S1. Confocal image of control IgG and p-cPLA2 in human platelets. The confocal image (10×100 magnification) of control IgG or p-cPLA2 in Tyrode's solution or collagen-activated platelets. Control IgG or p-cPLA2 was labeled with goat anti-rabbit IgG-conjugated FITC (shown in green color) as described in Materials and Methods.
